# Signaling pathways in brain tumors and therapeutic interventions

**DOI:** 10.1038/s41392-022-01260-z

**Published:** 2023-01-04

**Authors:** Shenglan Li, Can Wang, Jinyi Chen, Yanjie Lan, Weichunbai Zhang, Zhuang Kang, Yi Zheng, Rong Zhang, Jianyu Yu, Wenbin Li

**Affiliations:** Department of Neuro-Oncology, Cancer Center, Beijing Tiantan Hospital, Capital Medical University, Beijing, China

**Keywords:** CNS cancer, Target identification

## Abstract

Brain tumors, although rare, contribute to distinct mortality and morbidity at all ages. Although there are few therapeutic options for brain tumors, enhanced biological understanding and unexampled innovations in targeted therapies and immunotherapies have considerably improved patients’ prognoses. Nonetheless, the reduced response rates and unavoidable drug resistance of currently available treatment approaches have become a barrier to further improvement in brain tumor (glioma, meningioma, CNS germ cell tumors, and CNS lymphoma) treatment. Previous literature data revealed that several different signaling pathways are dysregulated in brain tumor. Importantly, a better understanding of targeting signaling pathways that influences malignant behavior of brain tumor cells might open the way for the development of novel targeted therapies. Thus, there is an urgent need for a more comprehensive understanding of the pathogenesis of these brain tumors, which might result in greater progress in therapeutic approaches. This paper began with a brief description of the epidemiology, incidence, risk factors, as well as survival of brain tumors. Next, the major signaling pathways underlying these brain tumors’ pathogenesis and current progress in therapies, including clinical trials, targeted therapies, immunotherapies, and system therapies, have been systemically reviewed and discussed. Finally, future perspective and challenges of development of novel therapeutic strategies in brain tumor were emphasized.

## Introduction

Brain tumors and other central nervous systems (CNS) tumors have a complicated classification according to histological and molecular findings. Based on the developments in the prior five publications from 1979, 1993, 2000, 2007, and 2016, and the recommendations of the Consortium to Inform Molecular and Practical Approaches to CNS Tumor Taxonomy (cIMPACT-NOW),^1–11^ the fifth edition of the World Health Organization Classification of Tumors of the Central Nervous System (WHO CNS5) in 2021 advance the role of molecular diagnostics in CNS tumor classification.^11^ Since the great development of cancer genomics revolutionizing the diagnostic criteria, the discovery of impactful and experimental molecular-targeted therapies provides new insights for current management and prognosis. In addition, the treatment of brain tumors suggests multi-disciplinary treatment (MDT) and individualized therapy to improve patients’ survival and quality of life.

This article briefly introduces the contemporary incidence, survival, as well as mortality of brain tumors and other CNS tumors, and focuses on the significant molecular signal pathway and currently considered therapeutic options (clinical trials, targeted therapies, immunotherapies, and system therapies) in glioma, meningioma, primary CNS lymphoma, and CNS germ cell tumors, setting by the WHO CNS5.

## Epidemiology of brain tumors

**Fig. 1:**
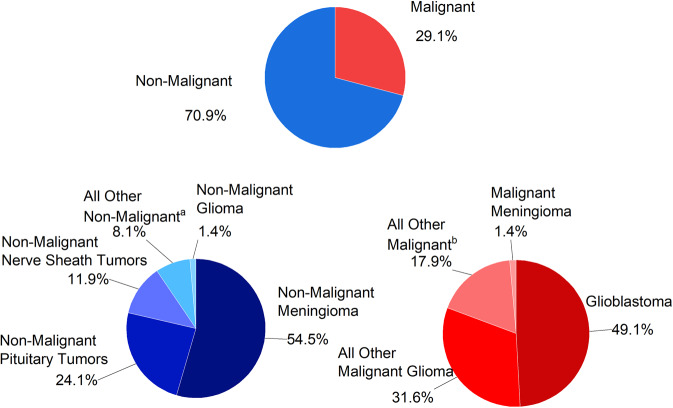
Distribution of Brain together with Other Central Nervous System (CNS) Tumors by Behavior and Major Histology Type, 2014 to 2018. **a** Includes histology with ICD-O-3 behavior code of /3 from neuronal and mixed neuronal–glial tumors, choroid plexus tumors, tumors of the pineal region, mesenchymal tumors, embryonal tumors, nerve sheath tumors, primary melanocytic lesions, lymphoma, germ cell tumors, tumors of the pituitary, other hematopoietic neoplasms, neoplasm unspecified, craniopharyngioma, hemangioma, as well as all other. **b** Includes histology with ICD-O-3 behavior code of /0 or /1 from tumors of the pineal region, neuronal and mixed neuronal–glial tumors, embryonal tumors, mesenchymal tumors, primary melanocytic lesions, craniopharyngioma, hemangioma, other tumors of cranial and spinal nerves, other hematopoietic neoplasms, germ cell tumors, neoplasm unspecified, as well as all other

Based on the data from the Central Brain Tumor Registry of the United States, the overall incidence of malignant brain tumors in patients of all ages decreased by about 0.8% per year from 2008 to 2017,^12^ while it has been elevated in non-malignant tumors.^13^ The brain tumors have a total incidence of 24.25/100,000, with 7.06/100,000 for malignant brain tumors and 17.18/100,000 for non-malignant ones between 2014 and 2018.^14^ Compared with 15 years ago (14.4/100,000), brain tumors’ overall incidence has almost doubled.^15^ By 2021, 88,190 new brain and other CNS tumors would be diagnosed in the U.S. population, including 25,690 malignant brain tumors and 62,500 nonmalignant brain tumors.^14^ Malignant brain tumors compromise no more than 1/3 of all brain tumors but are the causes of most disease deaths. The annual mortality rate is about 4.43/100,000, with an average of 16,606 annual deaths from primary malignant brain together with other CNS tumors,^14^ with gliomas accounting for 78.3% of malignant brain tumors and exceeding 50% of glioblastomas (GBM). Meningioma was the most frequent nonmalignant brain tumor, followed by pituitary tumors and nerve sheath tumors (Fig. 1).

## Incidence by age, gender, and race

The incidence of primary brain tumors varies by age, gender, and race. Both in malignant and nonmalignant brain tumors, the overall incidence in adults aged ≥20 years increased with age. In those aged ≥65 years, the incidence of most histological subtypes of brain tumors was highest, which was 1.5–8 times and 2–9 times higher in malignant brain tumors and non-malignant ones, respectively. However, for minors, the incidence rate of malignant brain tumors decreases with age, while it increases with age in non-malignant brain tumors. And the total incidence rate of brain tumors in people aged 0–4 years and 15–19 years is higher than that in children aged 5–14 years. Other gliomas and tumors (incidence rate:0.77/100,000) of the pituitary (incidence rate:0.88/100,000) were the main types.^14^

In terms of gender, malignant brain tumors (male: 8.28/100,000, female:5.98/100,000) were more easily seen in men than women, and the opposite was true for nonmalignant tumors (male: 13.07/100,000, female:20.97/100,000).^14^ For malignant brain tumors, gender differences gradually became apparent in adults aged ≥40 years. In adults aged ≥45 years, the gender differences were greatest, with 30% lower rates in females than males (ratio of female to male incidence rate, 0.69; 95% CI: 0.68–0.70).^12^ The incidences of GBM, diffuse astrocytoma, and other gliomas were higher in males than in females in the same age group. For nonmalignant brain tumors, gender differences gradually became apparent in adults aged ≥20 years. The gender difference was mainly in meningiomas and pituitary tumors. Especially for nonmalignant meningiomas, the incidence rate of females was about twice that of males in adults aged ≥65 years. However, the incidence of pituitary tumors in women decreased with increased age. It was currently believed that this was related to gender differences in lifetime exposure to endogenous hormones.^16^ In terms of race, the brain tumor total incidence rate in Black was the highest (24.58/100,000), which was the lowest (14.62/100,000) in American Indians/Alaska Natives. Among them, White had the highest morbidity of malignant brain tumors (7.55/100,000) and Black had the highest morbidity of non-malignant ones (20.14/100,000).^14^

## Survival of brain tumors

Between 1975–1977 and 2009–2015, the 5-year survival rate of all malignant brain tumors increased from 23 to 36%, with a greater increase in the younger age group.^12^ The 5-year relative survival rates for both malignant and nonmalignant brain tumors diagnosed by histology and age were revealed between 2009 and 2015. Overall, the 5-year survival rate was 35.6% for patients with malignant tumors, with GBM having the lowest rate at 6.6%.^14^ But the survival rate of a pilocytic astrocytoma can reach 94.4%.^14^ Additionally, the 5-year relative survival rates of people over 40 years old were far lower than that of people aged 0–14 and 15–39 years old.^14^ In contrast, the overall 5-year survival rate was as high as 91.8% for patients with nonmalignant tumors, with little difference between age groups.^14^

## Risk factors for brain tumors

The research on genetic and environmental risk factors of the brain and other CNS tumors has been continuous but without a breakthrough. To date, some gene loci and rare genetic mutations that may elevate the risk of some brain tumors had been identified.^17^ Some studies also focused on endogenous factors, such as allergy,^18,19^ head injury,^20^ and virus infection.^21,22^ Ionizing radiation remained the only well-defined environmental risk reason for brain tumors. Many studies had shown that low-dose therapeutic radiation can enhance the risk of many subtypes of brain tumors, including nerve sheath tumors, meningiomas, gliomas, and so on.^23–27^ Among them, it had the greatest impact on meningiomas.^24^ For glioma, young people were more vulnerable to ionizing radiation.^24^ However, the effect of diagnostic radiation exposure on brain tumors was uncertain.^23,28,29^ The influence of occupational exposure on brain tumors had been tested, but the results were inconsistent because of the small number of brain tumor cases and the difficulty in assessing individual exposure. Common carcinogens in occupational exposure, such as organic solvents, pesticides, and heavy metals (eg. lead, formaldehyde, and sulfur dioxide), had not been observed to link with brain tumors.^30,31^ Nearly 30–50% of cancers could be defended by appropriate nutrition habits, but their effects on brain tumors had not been fully explained. Foods rich in antioxidants (such as vitamins) and precursors of N-nitroso compounds (such as nitrite) were often considered to be closely related to brain tumors.^32–37^ The latest meta-analysis explored 12 food groups and found that tea and vegetables had a protective effect on glioma, while excessive intake of grains and processed meat significantly increased its risk.^38^ However, in the large prospective cohort study, the association between the single food group and brain tumors was not observed, while the Mediterranean diet pattern had a more significant impact on it from the perspective of the overall diet.^39^ Similar to occupational exposure, due to the limitations of dietary survey methods and regional differences in diet, most of the current studies were concentrated in Europe and America, lacking the research results of other populations such as Asia. The relationship between this two still needed to be further explored.

### Glioma

Glioma is a frequent primary brain tumor originating from glial cells. Based on the WHO CNS5 in 2021, gliomas are classified into adult-type diffuse gliomas, pediatric-type diffuse low-grade and high-grade gliomas (LGG and HGG), as well as circumscribed astrocytic gliomas.^11^ The localized gliomas often present benign biological behaviors that could be treated with complete surgical resection. Most diffuse gliomas are malignant and cured only by complete surgical resection. Grading using Arabic numerals is recommended, as highlighted by WHO CNS5.^11^ LGG comprises CNS WHO grades 1–2, whereas HGG comprises grades 3–4. LGG accounts for 6% of primary adult CNS tumors and usually has a good prognosis,^40^ but can recur and progress to HGG, especially grade 2 LGG.^41^ GBM accounts for 57% of all gliomas while 48% of primary CNS malignancies in HGG,^42^ have a median survival time of fewer than 2 years. Molecular changes and clinical significance in glioma are detailed in Table 1.

**Table 1:** Molecular changes in glioma and their clinical significance

Gene	Molecular alterations and pathway	Frequent subtype	Clinical significance	Ref.
IDH1/2	IDH1(R132), IDH2(R140), IDH2(R172) Catalyzing the conversion of α-KG to the R-2HG The FTO/m6A/MYC/CEBPA Signaling The mTOR Signaling The HIF1 Signaling Altered Epigenetics	The ~80% of grade II–III gliomas Secondary glioblastoma (GBM)	Better prognosis. Sensitive to the alkylating agent. Potential therapeutic targets. Diagnostically relevant.	^69,77,361^
MGMT	Promoter methylation The Wnt/β-catenin pathway Correlated with IDH mutation	The 30–60% of GBM	Better prognosis, in GBM. Sensitive to the alkylating agent. Potential therapeutic targets.	^362–365^
Chr 1p/19q	Codeletion Co-segregates with IDH mutations	The >80% of oligodendroglioma	Better prognosis. Sensitive to alkylating agent, in LGG. Diagnostically relevant.	^366–368^
EGFR	amplification EGFRvIII mutation (deletion of exons 2–7) The Ras/Mek/Erk Signaling The PI3K/AKT/mTOR Signaling The STAT3/5 Signaling The mTORC2/NF-κB Signaling The Notch Signaling	In GBM, 40–50% of the genes were amplified, including half of these genes with EGFRvIII mutation, and 10–20% of genes were overexpressed without amplification The 27% of IDH-wt LGG, does not exist in IDH-mut LGG	Potential therapeutic targets.	^84,86,90,93,96,369–374^
PTEN	mutations/deletions The PI3K/AKT/mTOR Signaling The sonic hedgehog signaling	The 41% of GBM The 23% of IDH-wt LGG, not exist in IDH-mut LGG	Poor prognosis. Potential therapeutic targets.	^84,371,375,376^
PIK3CA	mutation/amplification The PI3K/AKT/mTOR Signaling	The 13% of primary GBM The 9% of Secondary GBM The 20% of IDH-mut & 1p/19q no-codeletion LGG	Potential therapeutic targets.	^371,377–379^
PIK3R1	mutation/amplification The PI3K/AKT/mTOR Signaling	The 8% of GBM The 9% of IDH-mut & 1p/19q no-codeletion LGG	Potential therapeutic targets.	^371,378–380^
TERT	promoter mutation (C228T, C250T) Enhanced expression resulting from promoter mutation (90%) Mutually exclusive with ATRX loss The non-canonical NF-κB signaling, in C250T	The 50–74% of GBM The 68% of oligodendroglioma	Diagnostically relevant. Potential therapeutic targets. Contradictory reports in prognostically relevant.	^84,117,118,371,381–384^
CDK4/6 or CDKN2A/B or RB	CDK4/6 amplification CDKN2A/B loss RB1 mutation The retinoblastoma pathway	CDK4/6 amplification: The 7% of IDH-wt LGG The 15% of GBM CDKN2A/B loss: The 45% of IDH-wt LGG The 50% of GBM RB1 mutation: The 27% of IDH-wt LGG The 10% of GBM	Potential therapeutic targets.	^84,112,371,385–387^
BRAF	BRAFV600E point mutation BRAF Fusion Mutually exclusive The Ras/Mek/Erk Signaling	BRAF mutations: The 1.7% of GBM The >50% of epithelioid GBM The 60–70% of pleomorphic xanthoastrocytoma BRAF Fusion: The NA% of LGG	Potential therapeutic targets. Diagnostically relevant.	^84,119,388–392^
TP53 or MDM2 or MDM4	TP53 mutation/deletion MDM2 or MDM4 amplification The p53 pathway	TP53: The 28% of GBM The 14% of IDH-wt LGG The 94% of IDH-mut & 1p/19q no-codeletion LGG MDM2: The 7.6% of GBM MDM4: The 7.2% of GBM The 13% of IDH-wt LGG	Potential therapeutic targets.	^84,371,393–395^
MET	mutation/amplification Fusion overexpression The Ras/Mek/Erk Signaling The PI3K/AKT/mTOR Signaling The PKCδ/SRC/STAT3 Signaling The Wnt/β-catenin pathway	mutation/amplification: The 1.6% of GBM Fusion: The 7.6% of GBM overexpression: The 31.8% of GBM	Potential therapeutic targets. Poor prognosis.	^84,126,129,130,132,133,396^
PDGFRA	mutation/amplification The Ras/Mek/Erk Signaling The PI3K/AKT/mTOR Signaling	The 13.1% of GBM The 30% of DIPG	Potential therapeutic targets. poor prognosis	^84,386,397–400^
FGFR	FGFR alteration FGFR–TACC Fusion The Ras/Mek/Erk Signaling The PI3K/AKT/mTOR Signaling	FGFR alteration: The 3.2% of GBM FGFR–TACC Fusion: The 5% of GBM	Potential therapeutic targets. FGFR2 deletion: poor prognosis	^84,130,401–404^
NTRK1, NTRK2 and NTRK3	Fusion The MAPK Signaling The PI3K/AKT/mTOR Signaling	The 0.55–2% of glioma The >5.3% of pediatric HGG The 4% of DIPG The 40% of non-brainstem HGG with < 3-year-old	Potential therapeutic targets.	^405–410^
H3 K27	Alteration Altered Epigenetics The PDGFRA Signaling	The 78% of DIPG The 36% of non-brain stem DIPG	Potential therapeutic targets. Diagnostically relevant. Poor prognosis. H3.1-K27M with better prognosis.	^141,142,381,411–414^
Notch1	mutation/deletion Overexpression The Notch Signaling The NF-κB Signaling	The 31% of IDH-mut & 1p/19q no-codeletion LGG. Overexpression in GBM	mutation/deletion: Better prognosis. Overexpression: Poor prognosis. Potential therapeutic targets.	^371,415–419^
NF1	Mutation/deletion The Ras/Mek/Erk Signaling The PI3K/AKT/mTOR Signaling	The 10% of GBM The 20% of IDH-wt LGG	Potential therapeutic targets. In LGG: Poor prognosis.	^84,420–423^
ATRX	Mutation/deletion Mutually exclusive with 1p/19q codeletion genome instability	The 6% of GBM The 86% of IDH-mut & 1p/19q no-codeletion LGG	Potential therapeutic targets. Diagnostically relevant Better prognosis.	^84,371,424,425^
CIC	Mutations	The 62% of IDH-mut & 1p/19q codeletion LGG	Diagnostically relevant	^371,426^
FUBP1	Mutations	The 29% of IDH-mut & 1p/19q codeletion LGG	Diagnostically relevant	^371,427^

### Standard treatment of glioma

Although the new version of tumor classification has brought more advantages and significative guidance for clinical practice, it is currently not fully implemented in clinical application. Therefore, this review is based on the grading of gliomas.

For HGG, such as GBM, subtotal gross total resection, concomitant temozolomide (TMZ) radiochemotherapy at a dose, local radiotherapy to the tumor site, and tumor treating fields should be considered as standard treatments.^43–46^ All GBM will finally progress or relapse to recurrent GBM (rGBM), while without standard treatment. In addition, bevacizumab, known as an anti-vascular endothelial growth factor (VEGF) antibody, showed improved progression-free survival (PFS) in GBM. Bevacizumab has been applied for rGBM with the approval of the US Food and Drug Administration (FDA).^47,48^ LGG correlates with a molecular phenotype, and oligodendrogliomas with IDH-mut and 1p19q codeletion possess the best prognosis, and then those with IDH mut and 1p19q intact, while those with IDH wild type have the worst prognosis. Therefore, the patients should be surgically removed as quickly to avoid subsequent malignant tumor progression, while accurate recognition of the molecular subtype of the tumor is very essential for LGG.^49^ For high-risk LGG, surgical treatment alone is not sufficient, and local postoperative radiotherapy should be administered at 50–54 Gy, accompanied by six cycles of adjuvant procarbazine/lomustine/vincristine (PCV).^50^ Carboplatin and vincristine are regarded as the standard treatment for some unresectable children with LGG.

### Molecular targeted therapy

O6 methylguanine DNA methyltransferase (MGMT) is a repair protein^51,52^ that is encoded by the MGMT gene, which can reverse DNA alkylation by depleting itself. TMZ, the standard therapy of GBM, is known as an alkylating agent that evokes tumor cell death through DNA alkylation at many sites. In patients with MGMT promoter methylation found in 30–50% of isocitrate dehydrogenase (IDH)-wt GBM,^53^ gene promoter methylation would repress the expression of this gene. Therefore, with MGMT promoter methylation, glioma patients benefit more from treatment with TMZ.^51,54^ However, a discordance of MGMT promoter methylation with protein expression was detected in various patient.^55,56^ This may be related to the regulation of MGMT protein by Wnt signaling in addition to the regulation of MGMT promoter methylation.^57^ Furthermore, MGMT methylation predicts longer survival at diagnosis, while this was not the case at relapse,^58^ and presumably, TMZ resistance was also associated with rearrangement mutation or MGMT gene fusion.^59^ Therefore, it is a reasonable strategy to treat TMZ-resistant glioma patients by developing targeted MGMT-sensitizing TMZ. A phase I trial (NCT01700569) demonstrated that the combination of TMZ, folic acid, as well as radiotherapy was feasible to promote MGMT methylation in patients with unmethylated MGMT.^60^ In addition, a preclinical study showed that bortezomib can strengthen the GBM’s sensitivity to TMZ by decreasing MGMT levels.^61^ These suggest that targeting MGMT induces TMZ sensitivity is very promising. According to Kingson Lin et al.,^62^ mismatch repair (MMR)-independent cell killing can be induced selectively in MGMT-depleted tumors to overcome resistance mechanisms. The agents deposit a kind of dynamic DNA lesion, which can be reversed by MGMT. However, in MGMT-deficient settings, it slowly evolves into an interstrand cross-link, leading to MMR-independent cell death with low toxicity both in vitro and in vivo. This finding may bring new therapies for gliomas and may offer a novel paradigm for the design of chemotherapeutic agents for exploiting specific DNA repair defects.

Mutation of IDH results in altered IDH enzymatic activity, and mutant IDH1 with novel enzymatic activity can generate R-2-hydroxyglutarate (R-2HG).^63^ The R-2HG alters GBM epigenetics by inhibiting the catalytic activity of tet methylcytosine dioxygenase (TET2), which the a-KG-dependent dioxygenases catalyze the hydroxylation of 5-methylcytosine into 5-hydroxymethylcytosine.^64^ Accordingly, IDH1^R132H^ mutation triggers the CpG island hyper-methylator phenotype in gliomas.^65,66^ DNA methylation results in the gliomas’ development by enhancing the number of stem cells and impairing differentiation.^67,68^ Interestingly, the anti-tumor potencies of R-2HG in impeding proliferation/survival of fat mass and obesity-associated (FTO)-high cancer cells via modulating the FTO/m6A/MYC/CEBPA signaling.^69^ Moreover, the DNA repair activity of mammalian alkylation protein B homolog 2 (ALKBH2) and alkylation protein B homolog 3 (ALKBH3) could reverse alkylation on 1meA and 3meC,^70,71^ which is restricted by R-2HG in vitro^72^ Importantly, the production of R-2HG makes IDH mutant cells sensitive to alkylating agents.^72^ The clinically significant bifunctional alkylating agents procarbazine and CCNU/lomustine induces highly genotoxic DNA interstrand crosslinks, and are a part of the PCV chemotherapeutic regimen successfully utilized in combination with radiotherapy for the treatment of brain tumors with IDH mutation status.^73^ In addition, the mutant IDH1/2 and R-2HG exhibit control mechanistic targeted of rapamycin (mTOR) and hypoxia-inducible factor-1 (HIF1) Signaling.^74–76^ Since the mechanisms and clinical implications remain to be clarified and have been discussed in excellent reviews,^77^ they will not be described here. Although IDH mutation predicted a better clinical prognosis and GBM patients who CNS5 were all IDH-wt. However, there are astrocytomas grade 3–4 IDH mutant and grade 3 IDH mutant oligodendrogliomas. In other tumors, it was found that the efficacy of Ivosidenib targeting IDH was significant (NCT02074839, NCT02677922).^78–81^ Ivosidenib showed good tolerability and efficacy in patients with recurrent or progressive IDH-must gliomas (NCT02073994).^82^ In addition, a vaccine targeting the IDH1 (R132H) mutation showed good tolerability with a high pseudo-progression rate for newly-diagnosed grade 3–4 gliomas (NCT02454634).^83^ Due to successful attempts in other tumors, many clinical trials targeting IDH mut gliomas are being initiated (NCT02771301, NCT04906473).

Epidermal growth factor receptor (EGFR) is a common site of oncogenic mutation in IDH-wt GBM^84^ and has participated in tumor cell proliferation, migration, and escape.^85^ About 50% of GBM samples have EGFR mutations, of which more than 40% have gene amplification, and the rest consists of gene mutations, rearrangements, etc.^84,86–88^ EGFR variant III (EGFRvIII) (deletion of exons 2–7), as the most significant gene mutation of EGFR, leads to an in-frame deletion variant with a truncated extracellular domain with ligand-independent constitutive activity.^84^ EGFRvIII induces mTORC2 kinase activity, which is partially restricted by phosphatase and tensin homolog (PTEN). The mTORC2 signaling enhances GBM growth and survival and subsequently activates nuclear transcription factor-kappa B (NF-κB). Moreover, this mTORC2-NF-κB pathway makes cells and tumors of GBM resistant to chemotherapy in a manner independent of V-akt murine thymoma viral oncogene homolog (AKT).^89^ Furthermore, the EGFRvIII and wild-type EGFR strongly activate the RAS/MEK/ERK signaling, the PI3K/AKT/mTOR signaling, the Notch signaling, and the signal transducer and activator of transcription (STAT) 3/5 signaling.^90–92^ These signalings functions in the regulation of cell activities.^91^ This is one of the grounds for targeting these signaling pathways to treat GBM, which will be elaborated on later.

There are usually two strategies to target EGFR for GBM treatment: EGFR inhibitors, antibodies, vaccines, chimeric antigen receptor-T (CAR-T) cells, and other therapies to reduce the level of EGFR overexpressing cells. Gefitinib and dacomitinib, as EGFR inhibitors, were not effective in the EGFR-amplified GBM patients (NCT01520870, and NCT02447419),^93,94^ which may be caused by the low permeability of the blood–brain barrier. However, Osimertinib, a third-generation EGFR inhibitor, has a better blood–brain barrier permeability.^95^ Preclinical studies have revealed that Osimertinib regulates the mitogen-activated protein kinase (MAPK) pathway and then inhibits the transcription factor EGFR-transcriptional co-activator with PDZ-binding motif (TAZ) to inhibit GBM-patient-derived xenografts (PDX) model.^96,97^ However, its specific clinical effect remains to be studied.

EGFR antibodies have mostly failed in clinical trials for glioma therapy.^98,99^ Nevertheless, nimotuzumab is more useful in GBM patients with the activated AKT/mTOR signaling pathway.^100^ In addition, depatuxizumab mafodotin, an antibody–drug coupling drug, is effective for rGBM that relapses after TMZ standard treatment^101,102^ but is ineffective in newly diagnosed GBM (NCT02573324).^103^ In response to rGBM harboring EGFRvIII mutations, the vaccine rindopepimut in combination with TMZ demonstrated efficacy (NCT00458601)^104^ but failed to exhibit efficacy in phase III clinical trial (NCT01480479),^105^ see immunotherapy section below. CAR-T regimen is still in phase I trials and has shown the expected effects (NCT02209376).^106,107^

PI3K/AKT/mTOR is a frequent mutation pathway in IDH-wt GBM patients.^108^ In particular, mutations in PTEN and PIK3K genes^108^ lead to abnormal activity of the PI3K/AKT/mTOR pathway, promoting GBM cell viability, stem cell maintenance, and tumor formation.^109^ This may be linked to the complex and extensive molecular modulation of PI3K/AKT/mTOR. Therefore, the method of improving patients’ tolerance to higher doses to ensure the effect of targeted therapy needs to be proposed urgently.

Like the tumor-suppressor gene tumor protein p53 (TP53) gene, the retinoblastoma tumor suppressor protein (pRB) pathway is very important in the regulation of the cell cycle.^110,111^ In most of the IDH wild-type GBMs, there are homozygous deletions of cyclin-dependent kinase inhibitor 2 A/B (CDKN2A/B), amplifications of cyclin-dependent kinases 4 and 6 (CDK4/6), and alterations in the RB1 gene in the pRB pathway.^84,112^ The CDK4/6 form the common functional heterodimeric complexes with cyclin D1-3 (cycD1-3), which can phosphorylate and inactivate the RB protein.^113^ Inactivation of RB deregulates negative modulation of the E2F transcription factors, thereby inducing the G1/S transition in the cell cycle, thus allowing DNA synthesis and cell growth.^113^

However, the efficacy of the CDK4/6 inhibitor palbociclib in the treatment of GBM is disappointing (NCT01227434). Clinical trials of another CDK4/6 inhibitor, ribociclib, also showed limited efficacy (NCT02345824).^114^ In addition, clinical trials of TG02 targeting CDK9 in rGBM therapy and newly diagnosed GBM are ongoing (NCT02942264, NCT03224104).

Telomerase reverse transcriptase (TERT) can maintain the length of telomeres and promote the immortality of tumor cells. The TERT promoter mutations can produce new E-26 transcription factor binding sites, promote transcription, and thus increase activity.^115^ Interestingly, the combination of the E-26 transcription factor and the mutant TERT promoter is not enough to drive its transcription, but this process requires the non-canonical NF-κB signaling to stimulate a response, and continuous telomerase activity, leading to cancer progression.^116^ TERT promoter mutations are very common in the GBM of IDH-wt.^84^ However, in the current clinical trials, it has not been studied as a target for GBM therapy. However, preclinical studies have shown that inhibiting TERT activity can prolong the survival of GBM mice.^117^ In addition, publications have shown that inhibiting the TERT activity of GBM can sensitize TMZ.^118^ Therefore, targeting TERT to treat GBM is a worthy strategy for further study.

V-raf murine viral oncogene homolog B1 (BRAF) is implicated in the MEK/ERK signaling pathway activation and promoting cell proliferation.^119^ Targeted BRAF mutations, especially BRAFV600E missense mutations, have shown remarkable efficacy in other tumors.^120^ Although BRAF mutations have been observed in diverse glioma subtypes, they are rare in HGG.^121^ BRAF’s low mutation rate in HGG limits the therapeutic effect.^122^

P53 as a tumor suppressor, mouse double minute 2 and 4 (MDM2 and MDM4) as negative modulators of p53 protein, is one of the most frequent mutation sites in glioma.^108,123^ P53 can block the cell cycle arrest and induce apoptosis in G0/G1 in response to genotoxic stress.^124,125^ Mutational inactivation of TP53 and censored inactivation of MDM2/4 promote uncontrolled proliferation of glioma cells.^84^ The interaction between hepatocyte growth factor receptor (MET) and hepatocyte growth factor (HGF) contributes to auto-phosphorylation at diverse tyrosine residues, thereby resulting in the recruitment and activation of many signaling effectors, such as growth factor receptor-bound protein 2 (GRB2), GRB2-associated binding protein 1 (Gab1), Steroid receptor coactivator (SRC), SRC homology collagen (Shc), SRC homology region 2 (SH2)-containing protein tyrosine phosphatase 2 (SHP2), phospholipase C-gamma (PLC-γ), focal adhesion kinase (FAK), and casitas B lineage lymphoma (c-Cbl), along with the subsequent phosphorylation of downstream transducers, including PKCδ/SRC/STAT3, PI3K/Akt, Ras/MAPK/ERK, and Wnt/β-catenin pathway.^126–128^ Approximately 30% of GBM patients are characterized by MET gene fusion and high expression,^129,130^ which is considered to function in the drug resistance, recurrence, and migratory and invasive capabilities of glioma cells, especially in angiogenesis, radiation resistance, as well as hypoxia.^126,131^ However, rilotumumab,^132^ onartuzumab (NCT01632228),^133^ and cabozantinib (NCT00704288, NCT01870726)^134–136^ targeting MET have limited efficacy in the treatment of GBM.

Trimethylation of lysine 27 on histone H3 (H3K27) alteration occurs in 80% of pediatric diffuse midline gliomas (pDIPGs) and is a driving event leading to tumor initiation and progression.^137–140^ The H3K27 alteration elevates the activity of histone deacetylases (HDACs), and HDAC inhibitors are a potent compound for the reduced survival of pDIPG cells.^141,142^ A clinical trial of the HDAC inhibitor panobinostat for the treatment of pDIPG is ongoing (NCT02717455). In addition, synthetic peptide vaccines directed against the H3.3K27M epitope for the treatment of newly diagnosed DIPG patients and other H3.3K27-positive glioma clinical trials are ongoing (NCT02960230).

The vascular endothelial growth factor receptor (VEGFR) signaling pathway has been considered a key factor in GBM tumor survival.^143^ Meanwhile, GBM is featured with abnormal vascular proliferation. VEGF is upregulated in GBM and stimulates abnormal proliferation of tumor vessels by activating the essential downstream signaling pathways, such as MAPK/ERK1/2, endothelial nitric oxide synthase (eNOS), as well as mTOR.^144^ Interestingly, vessel normalization increases tumor blood perfusion and contributes to improved GBM patient survival (NCT00305656).^145^

Bevacizumab inhibits angiogenesis by acting as a humanized monoclonal antibody against the VEGF-A ligand,.^146^ A phase III trial of bevacizumab was shown to significantly improve PFS for newly diagnosed GBM and rGBM (NCT00884741) but did not significantly impact overall survival (OS).^47,147^ Bevacizumab treatment of GBM patients with IDH1-wt showed prolongation of OS (NCT00943826).^148^ The combination of bevacizumab and TMZshowed excellent efficacy and tolerability in recurrent/progressing GBM.^149^ In addition, bevacizumab in combination with CCNU and radiotherapy also alleviated PFS in patients with IGS-18 or “classical GBMs”.^150^

The transforming growth factor beta (TGF-β) protein family has complicated functions in diverse regulatory pathways,^151,152^ where TGF-β2 is a T cell inhibitor in the GBM tumor microenvironment^153^ that is found in approximately 90% of GBM tumor cells. Nevertheless, TGF-β1 receptor kinase inhibitor Galunisertib in combination with lomustine has limited efficacy in rGBM therapy (NCT01582269).^154^ Trabedersen, a TGF-β2-specific antisense oligonucleotide, is helpful for HGG treatment, particularly in patients with Karnofsky Performance Status (KPS) above 80% and under the age of 55 years (NCT00431561),^155^ but the overall effect is much lower than expected. Recent experiments have noted that TGF-β correlates with TMZ resistance and MGMT expression.^156^ Thus, TMZ and TGF-β combinations of inhibitors are promising.

Wingless and int-1 (Wnt) signaling modulates the neural progenitor cell (NPC) self-renewal, proliferation, as well as differentiation in the brain at varying stages of CNS development. GBM along with other cancers (e.g., digestive system) is associated with aberrant Wnt pathway activity,^157^ and in particular, GBM of the mesenchymal type is most active.^158^ But abnormalities in vital components of the Wnt pathway are not common in GBM.^159^ Preclinical studies have found that inhibiting the activity of the Wnt pathway inhibits TMZ-induced autophagy, which in turn promotes TMZ re-sensitization.^160^ However, clinical trials targeting Wnt signaling pathway for glioma treatment are currently lacking. The potency of the Wnt signaling pathway in GBM also still needs more investigation.

**Fig. 2:**
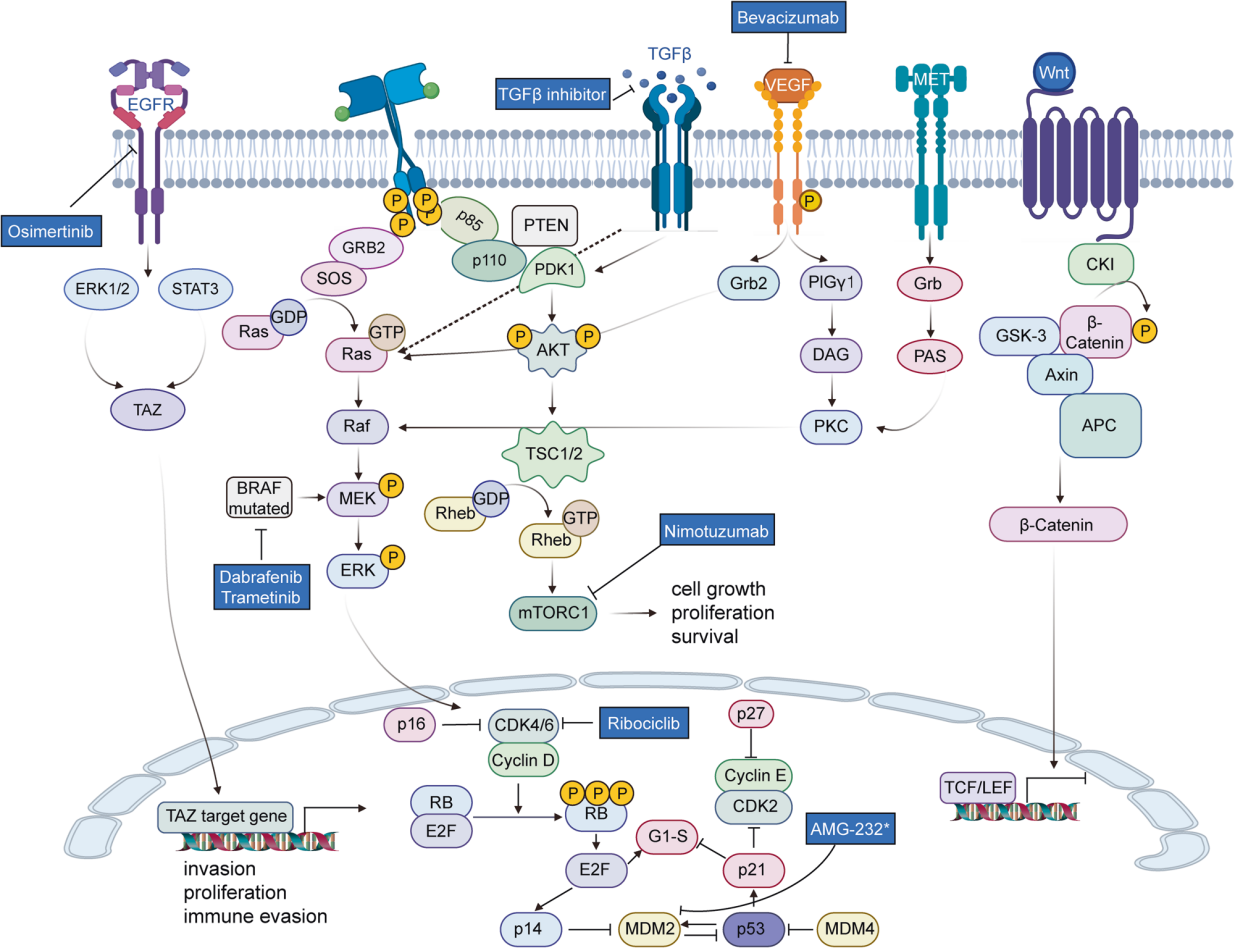
Signaling pathways, genetic mutations, as well as targeted treatment are implicated in glioma development. Tumorigenesis in glioma is activated through diverse mechanisms. Molecular alteration with clinically significant (such as MGMT, IDH, BRAF) eventually converges to several crucial signaling pathways (EGFR, PTEN, VEGF, MET, PI3K/AKT/mTOR, WNT, and TGF-β), resulting in tumor proliferation, invasion, cell survival, and immune evasion. “P” labeling is attached to the molecules that are phosphorylated due to signaling transduction. Created with BioRender.com (https://biorender.com) and Reactome pathway database (https://reactome.org/)

The above crucial signaling pathways involved in glioma were demonstrated in Fig. 2.

### Immunotherapy

Given the limited effect of standard treatment for gliomas (especially GBM) on survival, immunotherapy may be the future of GBM treatment. However, many difficulties and challenges exist for current immunotherapy of gliomas due to the special immune privileged status of the CNS, the low mutational burden of gliomas themselves, and the presence of a highly immunosuppressive microenvironment, which also implies the great potential of immunotherapy for gliomas.^161–163^ So far, many attempts have been made to develop immunotherapeutic approaches for gliomas, looking for therapeutic potential in immune checkpoint therapy, immune cell therapy, vaccines, oncolytic virotherapy, and other modalities. We will review these potential therapies and the immunological basis underlying glioma.

Immune checkpoint inhibitors (ICIS) have exhibited efficacy in many different clinical trials of malignancies, including in both adjuvant and neoadjuvant settings, accompanied by an overall long-term effect.^164–167^ Programmed cell death-1 (PD-1) interacts with programmed cell death ligand 1 (PD-L1). The combination of PD-1/PD-L1 reduces T cell receptor (TCR) and CD28 signaling, suppressing T cell effector activity and driving the immunosuppressive environment development.^168–170^ Although GBMs express elevated levels of PD-L1,^171^ to date, PD-1/PD-L1 immunotherapy trials using GBM have not been fruitful.^48^ The possible reason for this is that TMZ used in the trial affects PD-L1 level in GBM^172,173^ and is associated with suppression of immunity by dexamethasone.^174,175^

Cytotoxic T-lymphocyte-associated antigen 4 (CTLA-4) stimulates negative costimulatory signaling on the activated T cells,^176^ supporting an immunosuppressive environment by inducing immune tolerance.^177^ Currently, the CTLA-4 blocker ipilimumab is being assessed in GBM (NCT04323046, NCT04396860, and NCT04817254). A phase I exploratory cohort of the checkmate143 trial (NCT02017717) has demonstrated that ipilimumab plus nivolumab is safe.^178^ It remains to see whether CTLA-4 inhibitors will bring long-term advantages over the current standard of treatment.

**Fig. 3:**
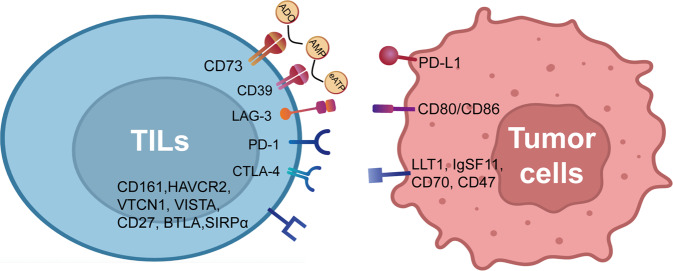
Current immune checkpoint molecules. Given the complex relationship between tumor cells and tumor-infiltrating lymphocytes (TILs), various clinical trials have been implemented based on the immune checkpoint molecules (PD-1/PD-L1, CTLA4, LAG-3, and other classic immune checkpoint molecules). Created with BioRender.com (https://biorender.com) and Reactome pathway database (https://reactome.org/)

Other immune checkpoints or immune-related molecules implicated in glioma include lymphocyte activation gene 3 (LAG-3), ecto-5’-nucleotidase/cluster of differentiation 73 (CD73), cluster of differentiation 161 (CD161), hepatitis A virus cellular receptor 2 (HAVCR2), indoleamine 2,3-dioxygenase 1 (IDO1), V-domain immunoglobulin suppressor of T cell activation (VISTA), V-set domain containing T cell activation inhibitor 1 (VTCN1), CD27/CD70, B, and T lymphocyte attenuator (BTLA), cluster of differentiation 39 (CD39), CD276, cluster of differentiation 47 (CD47), and many others. These target molecules are in clinical trials or only preclinical studies, which have been elaborated on in excellent reviews.^179^ Major immune checkpoint molecules were shown in Fig. 3. The clinical trials of immune checkpoint inhibitors in progress were shown in Table 2.

**Table 2:** The clinical trials of immune checkpoint inhibitors

Number	Treatment	Type of Study	Setting	N of patients
NCT02337686	Pembrolizumab	Phase II	Recurrent glioblastoma (rGBM)	20
NCT02667587	Nivolumab	Phase III	Newly diagnosed MGMT-methylated GBM	716
NCT02617589	Nivolumab	Phase III	Newly diagnosed MGMT-unmethylated GBM	560
NCT03047473	Adjuvant avelumab	Phase II	Newly diagnosed GBM	30
NCT02852655	Neoadjuvant pembrolizumab	Phase I	Surgically accessible recurrent/progressive GBM	25
NCT02974621	Cediranib	Phase II	rGBM	70
NCT03197506	Neoadjuvant pembrolizumab combined with adjuvant RT/TMZ/pembrolizumab	Phase II	Newly diagnosed GBM	50
NCT03158389	Matches one of 7 drugs to patients (APG101, idasanutlin,alectinib, vismodegib, atezolizumab, Palbociclib, and temsirolimus) in view of molecular markers after surgery.	Phase I/II	MGMT-unmethylated GBM	350
NCT03174197	Atezolizumab	Phase I/II	Newly diagnosed GBM	80
NCT03925246	Nivolumab	Phase II	Recurrent IDH mutant GBM	43
NCT03341806	Avelumab	Phase I	rGBM	13
NCT03426891	Pembrolizumab	Phase I	Newly diagnosed GBM	21
NCT04323046	Ipilimumab/nivolumab+adjuvant nivolumab	Phase I	Recurrent/progressive high-grade glioma	45
NCT03532295	Epacadostat+INCMGA00012	Phase II	rGBM	55
NCT03718767	Adjuvant nivolumab	Phase II	IDH mutant glioma	95
NCT03899857	pembrolizumab	Phase II	newly diagnosed GBM	56
NCT03493932	Nivolumab with BMS-986016	Phase I	rGBM	20
NCT03961971	MBG453 + Spartalizumab	Phase I	rGBM	15
NCT04047706	BMS 986,205+ nivolumab	Phase I	Newly diagnosed GBM	30
NCT04145115	ipilimumab+nivolumab	Phase II	Somatically hypermutated glioblastoma	37
NCT04225039	schedule	Phase II	rGBM	32
NCT04826393	ASP8374 + cemiplimab	Phase Ib	Recurrent high-grade glioma	24
NCT04396860	ipilimumab nivolumab	Phase II/III	Newly diagnosed IDH wild type MGMT-unmethylated glioblastoma.	485
NCT04608812	OS2966	Phase I	Newly diagnosed GBM	24
NCT04729959	Tocilizumab±atezolizumab	Phase II	rGBM	12
NCT04817254	ipilimumab	Phase II	Newly diagnosed GBM or gliosarcoma	48
NCT04656535	AB154 + AB122	Phase 0/I	rGBM	46
NCT04922723	daratumumab	Phase I/II	Newly diagnosed GBM	16
NCT04952571	Camrelizumab+ bevacizumab	Phase II	rGBM	94

#### Tumor-specific antigen polypeptide vaccines

Tumor-specific antigens refer to antigens that are expressed only by tumor cells, but not by normal tissues. EGFRvIII, introduced in the targeted therapy section, is a tumor-specific antigen. The vaccine peptide rindopepimut was synthesized according to the small amino acid sequence around the fusion site on EGFRvIII.^180^ This vaccine peptide has shown excellent safety and efficacy in both the phase I and phase II clinical trials.^104,181^ However, the difference between patients receiving the rindopepimut vaccine and those receiving the placebo vaccine could not be reproduced in phase III clinical trial.^105^ The possible reason is that rindopepimut selected out GBM cells with unmutated EGFR, leading to tumor recurrence. A phase II clinical trial of rindopepimut plus bevacizumab for recurrent GBM elucidated that patients receiving a peptide vaccine presented a better overall response rate and a longer OS, and could discontinue corticosteroid therapy more frequently than those treated with a placebo.^182^

#### Innate immune cell therapies and vaccines

Within the glioma immune microenvironment, innate immune cells are the key component. Glioma-associated innate immune cells mainly include tumor-associated macrophages (TAMs), myeloid-derived suppressor cells (MDSCs), and tumor-infiltrating dendritic cells (TIDCs). TAMs mainly consist of a small number of resident microglia and diverse bone marrow-derived macrophages.^183–185^ Microglia comprise only a small fraction of TAMs, which offers essential stimuli for tumors to allow for tumor progression.^186^ These TAMs were previously regarded as M2 immunosuppressive phenotype, but recent articles have indicated that they are a connection of M1 and M2 phenotype.^183,187–189^ TAMs maintain GBM’s mesenchymal phenotype via multiple mechanisms, which promote tumor growth and increase tumor aggressiveness.^184,190^ MDSCs can be assigned into 3 categories: CD15^+^ neutrophils, CD14^+^ monocytes, as well as CD15^−^ & CD14^−^ immature cells. MDSCs can stimulate T cell dysfunction through multiple mechanisms.^191,192^ GBM patients have elevated MDSCs in the blood, whose infiltration into the TME strengthens immunosuppressive effects.^191–194^ TIDCs are characterized by reduced antigen presentation and elevated expression of regulatory ligands/receptors along with broad immunosuppression. Many preclinical models have unveiled that the dendritic cell (DC) activity can be elevated by supplementation with stimulatory cytokines; this may imply a potent role for DC in GBM treatment. DCs have been recently extensively developed as a cellular platform for delivering antigen-specific vaccines to GBM patients.

Chlorogenic acid can modulate the polarization of TAMs toward the M1 phenotype in GBM.^195^ A clinical trial of chlorogenic acid for GBM patients is ongoing (NCT03758014). Capecitabine, in a low-dose and time-dependent manner, could attenuate intratumoral MDSCs.^196,197^ Eleven patients were treated with diverse doses of capecitabine for 5–7 days pre-surgery for recurrent GBM, and low-dose capecitabine and bevacizumab were subsequently used as maintenance therapy. Initial reports suggested that circulating MDSC numbers decreased as time went on in patients treated with higher doses and inflammatory infiltrates (eg. CD8^+^ T cells and Natural Killer (NK) cells), increased in the TME according to flow cytometry.^197^ The cytomegalovirus (CMV)-derived antigen pp65 is a novel target for DC therapy. The pp65, together with other CMV antigens, is expressed in approximately 90% of GBM samples.^198,199^ Reap et al used a vaccine with CMV pp65-specific T cells and CMV pp65 RNA-loaded DCs for treating newly diagnosed CMV seropositive GBM patients and observed that DCs increase T cell polyfunctionality and that this polyfunctionality improves survival.^198^ Another common DC vaccine strategy is based on tumor antigen profiling, incubating patient-derived DCs with synthetic peptides and, given the heterogeneity of GBM, often including several targets.^200^ ICT-107 is a hexapeptide DC vaccine for GBM therapy, which consists of gp100, IL13Rα2, peptides human epidermal growth factor receptor 2 (HER2), tyrosinase-related protein 2 (Trp-2), melanoma-associated antigens (MAGE-1), as well as automatic ingestion monitor-2 (AIM-2), all of which are elevated in GBM and glioma stem cells (GSCs). But a phase II placebo-controlled trial focusing on the ICT-107 vaccine in 124 patients showed only an increased PFS by only 2.2 months in vaccinated patients compared with placebo, with no significant difference in overall survival.^200^ But there were indications that patients’ HLA-A1^+^ vs HLA-A2^+^ status and MGMT promoter methylation status had a significant impact on patients’ outcomes. Although these studies have shown mixed outcomes, the ability of DC vaccines to a patient’s tumor cannot be underestimated, and innate immune cell therapies are currently shown to have both the advantages of very low side effects and high specificity.

#### Adaptive immune cell therapy

Compared to non-GBM controls, GBM and other gliomas can isolate peripheral circulating T cells in the bone marrow, leading to relative lymphopenia.^201^ In addition, GBM can evoke the invading CD4^+^ and CD8^+^ T cell apoptosis via the Fas/FasL signaling.^202–204^ Tregs, by producing TGF-β and IL10, affect tumor immune escape, thereby reducing the capability of CD8^+^ T cells to respond to their cancer cells.^205^ GBM cells express chemokine (C–C motif) ligand 2 (CCL2), leukocyte-specific protein-1 (LSP-1), STAT3, HIF-1α, and IDO to enhance the activity and survival of Tregs within the TME.^206–211^

CAR-T is a genetically engineered T cell with an artificial receptor directed against the selected antigen.^212,213^ These cells can bind tumor-specific antigens, independent of the natural mechanisms of antigen presentation, contributing to full activation of CAR-T cells, with infiltration into the tumor performing effector functions. CAR-T cells have received FDA approval in diffuse large B cell lymphoma (DLBCL) and acute lymphoblastic leukemia (ALL).^214,215^ The most studied targets of CAR-T in GBM are HER2, EGFRvIII, as well as IL-13αR2, which have already been published in clinical trial results.^216–220^ O’Rourke, et al stated that EGFRvIII-directed CAR-T cells are effective and safe.^106^ Brown, et al used CAR-T cells targeting IL-13Rα2 in recurrent GBM patients. The results were dramatic, with complete regression of all lesions and the effect maintained for 7.5 months.^217^ This clinical trial is still ongoing (NCT02208362).

Similar to CAR-T cells, NK cells have recently been applied in preclinical models of cancer with therapeutic roles for their innate functions to recognize/attack abnormal self-cells and overlook healthy cells by MHC-I recognition.^221^ Chimeric antigen receptor engineered-Natural Killer (CAR-NK) cells can be generated by chimeric antigen receptor technology and injected like T cells to form a similar type of therapy, though more studies are needed.

### Oncolytic virotherapy

Oncolytic viruses utilize the natural capability of viruses to replicate and lyse cells in combination with the release of neoantigens and damage-associated molecular patterns following tumor cell lysis, thereby invoking a robust immune response in the cancer area that further kills the tumor.^222–225^ The most common are herpesviruses, reoviruses, poxviruses, adenoviruses, or Zika virus.^223,226–228^

Markert, et al assessed the impact of G207, which does not function outside of tumor cells by viral ribonucleotide reductase. The trial results exhibited a good safety and efficacy profile; 11/12 had a treatment response, mOS was 12.2 months, and 4/11 were still alive at the 18-month follow-up. A larger phase II clinical trial is in progress (NCT04482933).^229^ The results of phase I/II, single-arm research assessing the safety of G47∆, in Japanese adults with recurrent/progressive GBM, were reported by Tomoki Todo et al., showing a 1-year survival rate of 38.5% and the mOS of 7.3 months^230^ (UMIN-CTR Clinical Trial Registry UMIN000002661). Another phase 2 trial assessing the efficacy of G47∆ in residual or recurrent GBM was also reported by Tomoki Todo et al., showing a 1-year survival rate of 84.2% and the mOS of 28.8 months (from initial surgery) and 20.2 months (after G47∆ initiation)^231^ (UMIN-CTR Clinical Trial Registry UMIN000015995). PVSRIPO, as a recombinant poliovirus that recognizes differentiation cluster (CD155), is upregulated in GBM cells,^232,233^ and a phase I trial of PVSRIPO in recurrent GBM therapy disclosed that the overall survival of the test group was elevated in contrast to the historical control group (NCT04479241).^232^ In another important trial, dnx-2401 (an oncolytic adenovirus with tumor selectivity by inactivating the E1A gene), was utilized for GBM treatment, which prevents the virus from replicating in normal cells with a functional RB (retinoblastoma) signaling pathway.^222,234^ Its results showed a good safety profile and immunoreactivity.^222^ Excellent reviews exist detailing previous clinical trials.^223,226^

In addition, the history of glioma clinical trials also suggested that therapeutic strategies targeting single-target, single-pathogenic mechanisms often lead to failure due to GBM being highly plastic with redundant survival mechanisms. Therefore, while developing precision-targeted therapies, it is highly imperative to develop combination therapy strategies, and excellent reviews have detailed the importance of immune combination therapy.^225^ However, current research to develop optimized combination therapy strategies is challenging due to the unclear tumor mechanism and the existence of a large number of combinable post-permutation therapeutic strategies, and perhaps the development of big data technologies and intelligent experimental platforms can help the field.^235^ Perhaps the most pressing thing at the moment is when there is a waning of efficacy or an enhancement of toxicity when combining traditional with emerging therapies, as this can lead to the failure of clinical trials. For example, reduced recurrent GBM PD-L1 expression by TMZ may be associated with nivolumab treatment failure in rGBM;^173^ Immunosuppressive effects of dexamethasone disable immunotherapy (particularly PD-(L) 1 treatment).^175^ Therefore, an in-depth study of the interaction between traditional and emerging therapies is highly necessary before clinical trials.

### Meningiomas

Meningiomas are a primary intracranial tumor in adults, and this disease harbors an annual incidence rate of approximately 8.58 cases per 100,000 population.^40,236^ Incidence elevates with age, especially in those over 65 years.^40,237^ The overall proportion of WHO grade 1 was 80%, and about 20% are WHO grade 2 or 3.^238–241^ Among WHO grade 1/2 meningiomas, the incidence is 2.3 times higher in women than in men.^40^ Most patients with meningioma are cured by surgery and radiation therapy. Incomplete resection or aggressive histological features of the tumor may lead to disease recurrence.^242,243^ Unfortunately, effective drugs have not been observed to date for patients with meningiomas who do not respond to conventional surgery or radiation therapy.^243–245^

At present, the changes in key gene characteristics in meningioma are closely related to tumor recurrence and prognosis and can be used as a promising therapeutic target.^246–253^ Although the WHO classification and subtypes of meningioma are mainly based on histopathology, 2021 WHO classification is also used for meningioma classification in combination with molecular biomarkers.^241,245^ Identifying gene mutations and longitudinal heterogeneity of tumor tissues by high-throughput sequencing are helpful for postoperative risk assessment and prognosis guidance, thus achieving personalized treatment of meningioma.^246,254,255^ Here, we summarize recent advances and ongoing efforts in molecular-driven therapy for meningioma.

### Molecular characteristics and signaling pathway in meningioma

2021 WHO classification emphasizes that the criteria for defining atypical or anaplastic (WHO grade 2/3) meningiomas apply to any subtype. Choroid meningioma and clear cell meningioma are re-assigned as CNS WHO grade 2 due to their higher recurrence rate than other CNS WHO grade 1 meningioma. Considering that other invasive features appear in combination with papillary and rhabdoid structures, classification based on rhabdoid cytology or papillary structure alone is not recommended.^11,256^ Some clinical studies have associated changes in molecular characteristics with histological subtypes of meningioma, and some molecules can be utilized as prognostic biomarkers to guide treatment. The utilization of novel techniques, such as whole genome sequencing (WGS), whole exome sequencing (WES), and transcriptome analysis, can better describe the mutation of these tumors and identify druggable targets.^257,258^

**Fig. 4:**
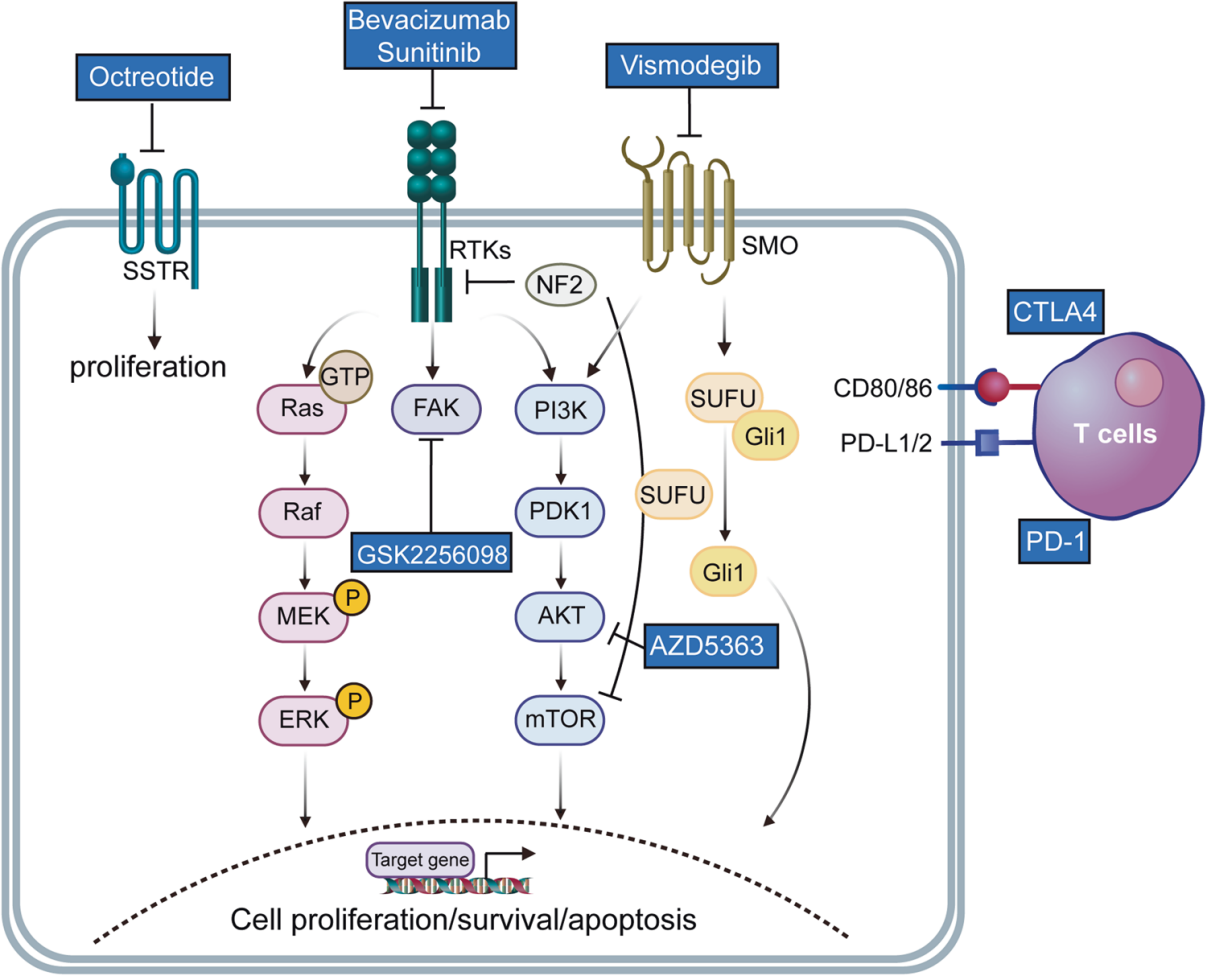
Summary of the activated signaling pathways and drug targets in meningioma. There are many cellular processes involved in meningioma growth, such as the PI3K–AKT–mTORC pathway, MAPK (mitogen-activated protein kinase) pathway, as well as the Hedgehog pathway. The figure shows the current medical therapies for meningioma, which target diverse molecular targets. Created with BioRender.com (https://biorender.com) and Reactome pathway database (https://reactome.org/)

It is common for atypical meningiomas to show multiple chromosomal gains as well as 1p, 6q, 10q, 14, and 18q chromosomal losses.^259^ Early studies identified 22q loss, including BAM22, breakpoint cluster region (BCR), and tissue inhibitor of metalloproteinase-1 (TIMP-1) as a common alteration in meningiomas.^259,260^ The early stages of meningioma tumorigenesis correlate to the inactivation of one or more genes from the 4.1 superfamilies, such as 4.1B (DAL-1) and neurofibromatosis-2 (NF-2).^259,261^ About 60% of sporadic meningiomas have the inactivation of NF2, which is closely related to disease recurrence.^262–264^ The alteration of NF2 is observed in different histological subtypes. For instance, 70% of fibroblastic and transitional meningiomas have NF2 mutations,^265–267^ but meningothelial, secretory, and microcystic meningioma are rare. In NF2 wild-type meningiomas, other common gene mutations were also associated with the classification and grading of meningiomas, such as AKT1, PIK3CA, tumor necrosis factor receptor-associated factor 7 (TRAF7), Kruppel-like factor 4 (KLF4), and smoothened (SMO).^252,268–272^ AKT1/TRAF7 and SMO mutations are representative markers of meningothelial meningioma.^273,274^ Secretory meningioma is often associated with KLF4 and TRAF7 gene changes.^275^ Nearly 10% of non-NF2 meningiomas harbor mutations in lysine-specific histone demethylase 5C (KDM5C), lysine-specific histone demethylase 6A (KDM6A), or SWI/SNF‐related matrix‐associated actin‐dependent regulator of chromatin subfamily B member 1 (SMARCB1), which encode epigenetic modifiers.^269^ SMO and AKT1-mTOR mutations are commonly seen in non-NF2, genomically stable meningiomas appearing in the skull base.^269^ The loss of histone H3K27me3 expression is closely related to meningioma recurrence.^276,277^ CDKN2A/CDKN2B (tumor suppressor genes on 9p21) loss of function is involved in meningioma progression from WHO grade 2 to grade 3, and TERT promoter mutation have been identified as a diagnostic marker for WHO grade 3 in the new WHO classification.^253,278–280^ In meningiomas, mutations in the Duchenne muscular dystrophy (DMD) gene have also been discovered,^258^ independently of TERT mutation status, and it was associated with worse clinical outcomes.^281^ Additionally, different subgroups of WHO grade 3 meningiomas have been identified with novel mutations. Other rarer germline mutations consisted of SWI/SNF Related, Matrix Associated, SMARCE1, (BRCA1-associated protein 1) BAP1, Actin Dependent Regulator of Chromatin, Subfamily B, Member 1 (SMARCB1), as well as a suppressor of fused (SUFU) genes. The BAP1 mutation was first described in rhabdoid meningiomas.^282^ BAP1 null cells rely on the enhancer of zeste homolog 2 (EZH2) for transformation, which is highly sensitive to EZH2 inhibition, thus opening new therapeutic perspectives.^283^ However, the work to translate molecular knowledge into clinical management is still ongoing. Consequently, genomics has enhanced our understanding of meningiomas’ molecular underpinnings, directing the way for further research into novel therapeutics. These molecular data from various individual studies were integrated into the activation of several signaling pathways in meningioma, as shown in Fig. 4.

### Treatment strategies for meningioma

Drug treatment for meningiomas is an option for patients who cannot undergo surgery or radiation therapy. Most of the better efficacy is still isolated cases and retrospective studies, and there is a lack of a large number of prospective clinical trial data support.^245^ The main problems in the clinical application of targeted drugs in meningioma include: how to reach the therapeutic target through the blood–brain barrier, how to avoid or reduce the side effects of drug therapy, and how to establish the evaluation criteria of therapeutic effectiveness. Currently, emerging trials of meningioma incorporating genomic information into criteria are expected to improve future clinical outcomes through precision medicine.^284^ As a common type of intracranial tumor, complete surgical resection is a standard treatment for meningioma. To avoid functional impairment, some patients failed to be successfully treated by surgery alone or completely resected safely. The treatment of refractory diseases is primarily related to WHO grade 2 and 3 meningiomas or inexcision.^285^ Several drugs are now being studied in clinical trials for meningiomas, focusing on cytotoxic agents,^286^ hormone agents,^287,288^ growth factor receptor antagonists,^289^ angiogenesis inhibitors,^290–292^ and immunotherapy.^293^ But no studies have revealed marked response, sustainable tumor control, or prolonged survival. Thus, if patients with meningioma fail to benefit from surgery or radiation, the disease becomes more difficult to treat. Recently, considerable achievements have been obtained in molecular gene research of meningioma, and the nature of recurrent refractory meningioma has been further revealed. For example, the expression of fatty acid synthase (FASN) is up-regulation in malignant meningioma, and inhibition of FASN can inhibit the proliferation of meningioma cells.^294^ Song et al. reported that FASN may be a target for malignant meningioma.^295^ The future diagnosis and treatment of meningioma based on molecular genes may bring more hope to malignant meningioma patients.

#### WHO grade 1 meningioma

For asymptomatic and sporadic meningiomas, regular MRI observation is the preferred strategy. For growing, symptomatic tumors, surgery is preferred.^296,297^ Surgical excision of tumor tissue was performed for histopathological and molecular pathological examination.^298^ In addition, surgery was evaluated based on Simpson grades of resection, which was used as a prognostic indicator of recurrence risk.^299,300^ Radiosurgery or fractionated radiotherapy may be used as an alternative to surgery.^301^ Currently, no useful drugs have been found for routine clinical treatment of WHO grade 1 meningioma. Pay attention to whether the patients have neurological and cognitive dysfunction to avoid affecting their quality of life. MRI evaluation is recommended periodically after observation or treatment.

#### WHO grade 2 meningioma

Surgical resection of WHO grade 2 meningiomas are preferred. Simpson I resection should be performed as closely as possible.^245,302^ When meningioma invades complex sites, it is difficult to avoid nerves, large vessels, and functional areas by surgery, and complete tumor resection may not be possible, resulting in an increased risk of recurrence. The follow-up time was shorter than that of WHO grade1 patients, usually 6 months, up to 5 years postoperatively.^245^ For patients with Simpson IV–V resection, tumor recurrence can be avoided or delayed by combining radiotherapy.^245^

#### WHO grade 3 meningioma

WHO grade 3 meningioma has a rapid growth rate, high recurrence tendency, and strong invasiveness, which can lead to systemic metastasis. It is recommended that surgical resection be as complete as possible, combined with fractional radiotherapy with a total dose of not less than 54Gy.^303^ Follow-up is followed at 3 months after initial treatment and 3 or 6 months thereafter.^303^ Drug therapy is in clinical trials and lacks data to support it.

#### Spinal meningiomas

Surgical resection is a preferred approach for spinal meningiomas. On the premise of not damaging the nerve function, the operation should achieve Simpson 1 resection as much as possible. Removal of the dura should not be the target for ventral spinal cord or severely calcified meningiomas.^304,305^ If surgery is not available or spinal cord decompression is not required, stereotactic radiosurgery or hypofractionated radiotherapy may be used instead.

#### Targeted therapies for meningioma

Currently, most clinical studies on meningioma are restricted by the small number of patients, the heterogeneity of tumor types and previous treatments, the lack of prospective controlled trials, and underpowered, resulting in the absence of high-grade evidence-based drugs for clinical use. A variety of therapeutic targets have been recognized for targeting the aforesaid genetic biomarkers in meningiomas, including VEGF/VEGFR, platelet-derived growth factors (PDGFs) and their receptors (PDGFR), EGFR, PIK3CA, mTOR pathway, progesterone receptor (PR), somatostatin (SST), PD-1/ PD-L1, etc. Studies have elucidated that VEGF is expressed in 84% of meningiomas, and the expression of VEGF elevates with the increase of meningioma grade.^306^ The VEGF inhibitor bevacizumab has shown clinical benefit in meningioma patients that are difficult to treat with surgery and radiotherapy.^291^ Sunitinib is a small-molecule tyrosine kinase inhibitor targeting VEGFR and PDGFR. For the treatment of malignant meningioma, a prospective, multicenter, single-arm Phase II clinical study on sunitinib demonstrated that 42% of patients did not develop tumorigenesis within 6 months.^307^ Besides, a recent study of the combination of the SST receptor antagonist octreotide and everolimus in recurrent meningiomas found that 6-month and 12-month survival rates were 90 and 75%, respectively. The growth rate of tumor volume reduced in 78% of patients after 3 months of treatment, a decrease of more than 50%. This article showed that the octreotide and everolimus combination had a better anti-meningioma activity.^308^ PD-L1 and other immune checkpoint inhibitors in phase II clinical trials are being evaluated in high-grade and recurrent meningioma patients. It is hoped that these immunotherapies will elucidate the efficacy of meningioma treatment.

Vismodegib, an inhibitor of the SMO enzyme, has been approved by the FDA for treating advanced basal cell carcinoma.^309^ Despite active research into TRAF7 and KLF4’s role in meningioma development, neither genetic alteration has been regarded as a potent therapeutic target. There is anecdotal evidence suggesting that AKT inhibitors are effective in meningiomas with AKT1 mutations.^251^ The tumor suppressor activity of NF-2 is modulated partly by eliminating the interactions with FAK signaling, and NF-2 inactivation or q22 deletion with tumor cells has been revealed to respond to FAK inhibition.^310^ GSK2256098, a FAK inhibitor, is currently found to function in the treatment of NF-2 mutation-associated meningiomas (NCT02523014). The completed and ongoing studies of meningioma in recent years are summarized in Table 3.

**Table 3:** Clinical trials of systemic treatment for meningioma

Treatment	Study type	Setting	N of patients	Results
Hydroxyurea	retrospective case series	recurrent WHO grade 1 meningioma	60	Duration of stable disease: 3–12 months (median 4.0 months)^286^
Hydroxyurea	retrospective case series	recurrent WHO grade 2/3 meningioma	35	6-month PFS: 3.0% (median PFS 2.0 months)^428^
Interferon-α	Phase 2	Recurrent grade 1	35	6-month and 12-month PFS: 54%, 31%; mOS: 8 months^429^
Interferon-α	Retrospective case series	Recurrent WHO grade 2/3	35	6-month PFS: 17%^430^
Bevacizumab	retrospective review	recurrent meningioma	14	6-month PFS: 86%^431^
Bevacizumab	retrospectively study	Atypical and anaplastic meningiomas	15	mPFS: 26 weeks. 6-month PFS: 43.8 %^290^
Mifepristone	Phase III	unresectable meningioma	164	Failure-free and OS were no statistical difference between mifepristone and placebo^288^
Pasireotide LAR	phase II	recurrent or progressive meningioma	34	It has limited efficacy in recurrent meningiomas^244^
Octreotide	phase II	recurrent high-grade meningioma	9	6-month PFS: 44.4 %, mPFS: 4.23 months^432^
Sandostatin LAR	prospective pilot trial	recurrent meningiomas	16	6-month PFS: 44%, mOS: 7.5 months^433^
Temozolomide	Phase II	refractory meningioma	16	Time to tumor progression: 2.5–5.0 months (median 5.0 months); OS: 4–9 months (median 7.5 months)^434^
Trabectedin	phase II	recurrent WHO grade 2 or 3 meningioma	90	not improve PFS and OS^435^
Octreotide and everolimus	phase II CEVOREM trial	recurrent meningiomas	20	6-month PFS: 55%, and OS 6- and 12-month were 90 and 75%, respectively^308^
Everolimus and bevacizumab	phase II	recurrent meningioma	18	median duration of disease stabilization: 10 months^436^
Sunitinib	phase II	recurrent WHO grades 2–3 meningioma	36	mPFS: 5.2 months, and mOS: 24.6 months^307^
Nivolumab	phase II	recurrent atypical/anaplastic meningioma	25	6-month PFS: 42.4%; mOS: 30.9 months; One patient achieved radiographic response (ongoing at 4.5 years).^293^

*PFS* progression-free survival, *OS* overall survival; progression-free survival, *mPFS* median progression-free survival, *mOS* median overall survival, *N* number

### Central nervous system germ cell tumors (CNS GCTs)

CNS GCTs are rare tumors that usually primarily affect in midline location of children and adolescents, and with a different tumor distribution demographically, account for no more than 4% of all primary CNS tumors in Western Europe and reach a high incidence of 11% in Asia, with a male predominance.^311–313^ Radiation therapy status was a vital predictor of death, and chemotherapy was also significant among all histological subtypes, even adjusting for age at diagnosis.^311^

Currently, there are very few studies on the molecular biology of CNS GCTs. CNS GCTs diagnosis is on account of the combination of clinical features, tumor markers, and neuroimaging features, and is confirmed by cytopathology and histopathology. However, the discovery of human chorionic gonadotropin (HCG) and alpha-fetoprotein (AFP) in CSF and serum is a great step forward for the diagnosis stage, treatment response, detection relapse, and estimated prognosis in intracranial germ cell tumors (ICGCTs).^313^

The origin of germinomas is still unclear. However, based on immunohistochemical staining and high throughput sequencing, DNA hypomethylation, MAPK, and/or PI3K pathway alterations, as well as chromosomal abnormalities, exhibit a triad implicated in the CNS GCT pathogenesis.

Germinoma cells recapitulate the characteristics of pluripotent human embryonic stem cells (PGCs) by elevating the genes responsible for self-renewal, including pluripotency factor Octamer-binding transcriptional factor 4 (OCT4), NANOG, and KLF4. In contrast, non-germinomatous germ cell tumors (NGGCTs) are featured with the levels of genes related to epithelial–mesenchymal transition, neuronal differentiation, or the Wnt/β-catenin pathway. While chromosomal instability is a characteristic of all CNS GCT, global DNA hypomethylation is only found in germinoma. Somatic tyrosine kinase receptor (KIT)/RAS and PI3K/AKT mutations have been identified in all CNS GCTs, especially germinoma.^314–316^

### CNS germinomas treatment

Germinomas are radiosensitive and high cure rate with radiotherapy (RT) alone; in retrospective and prospective series, the 5-year overall survival rates were above 80%.^317^ Chemotherapy (intensive cisplatin and cyclophosphamide-based chemotherapy) alone could achieve remissions, yet, the long-term outcome was unsatisfactory, including unacceptable morbidity and mortality.^318^ Thus, the standard germinoma system treatments contain chemotherapy (Carboplatin/Cisplatin and Etoposide ± Ifosfamide) and radiotherapy, to reduce the volume and dose of RT. As for the high radiosensitivity of germinomas, the surgical section is often used for hydrocephalus treatment and obtaining a histological diagnosis and is not necessary for extension tumor resection. Besides, surgical resection is different in the management of pediatric and adult populations. As for pituitary germinomas, the most comment treatment was radiation + chemotherapy in pediatrics, while radiation + gross total resection + chemotherapy in adults.^319^

For localized CNS germinomas, the treatment may include craniospinal irradiation (CSI) alone, chemotherapy, or reduced-field radiotherapy. The RT treatment is often applied for covering the whole ventricular (WV) system and is followed by primary tumor boost (PTB). However, optimal RT dosage and field inclusion remains controversial. In terms of radiotherapy techniques, passively scattered proton beam therapy (PSPT) provides lower doses of radiation to the healthy tissue around the tumor, and larger temporal lobe and hippocampal volumes were retained when compared to intensity-modulated radiotherapy (IMRT).^320^ For disseminated germinomas, chemotherapy and CSI combination are almost consistently recommended for patients with disseminated disease at diagnosis.^321^ For recurrent CNS germinomas, the salvage therapy consists of local or whole-axis RT, surgery, as well as myeloablative high-dose chemotherapy (HDC) with autologous hematopoietic stem cell rescue (ASCR).^322^

**Fig. 5:**
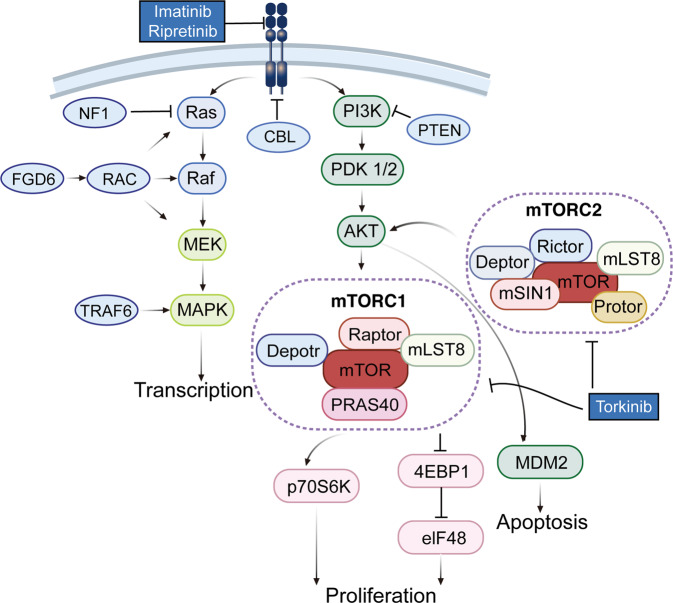
Signaling pathways involved in CNS germinomas pathogenesis and potential molecular targeted therapy. KIT and RAS mutations are the most frequent genes in CNS germinomas. Gain of function mutations of KIT proto-oncogene leads to the KIT protein activation, leading to the upregulation of MAPK or PI3K signaling pathway, which implicated tumor proliferation, migration, and apoptosis resistance. In addition, upregulated PI3K pathway causes a frequent mutation of the mTOR gene, which leads to cell growth via mTOR1 mutation and cell survival via mTOR2 mutation and AKT. Created with BioRender.com (https://biorender.com) and Reactome pathway database (https://reactome.org/)

With the development and application of high throughput sequencing technology, the genomic and epigenetic mechanisms of germinomas have been gradually revealed, and molecular targeted therapy is carried out by degree. The KIT mutation and mTOR mutation were confirmed in CNS germinomas and could be the potential target for therapy.^316,323^ However, no related KIT or mTOR pathway-targeted clinical trials have been recruited or carried out for CNS germinomas. Figure 5 showed potential targeted drugs for CNS germinomas according to the molecular profiles.

### CNS NGGCTs treatment

CNS NGGCTs are difficult to treat with conventional surgery and RT, and their total cure rate in the era of RT alone was 25%. The current treatment for CNS NGGCT is achieved by combining surgery, chemotherapy, and RT, using a wider field and a higher dose. For malignant NGGCTs, the purposes of treatment are to control the local tumors, with RT covering leptomeningeal tumor spread, as well as chemotherapy eliminating systemic tumor dissemination.^324^ The combination of surgery, chemotherapy and RT is tailored based on grouping and staging. Among them, the improved survival rate was closely linked to the extent of tumor resection. Metastatic disease is diagnosed by positive CSF cytology and/or distant drops in craniospinal MRI. These malignant GCTs present a high incidence of spinal metastasis or subarachnoid dissemination, which makes CSI with a high-dose local boost essential. Metastatic germinomas may be treated by craniospinal irradiation.^325^ Chemotherapy is a vital component of multi-modal treatment, while chemotherapy-only strategies are not advised because of the high local treatment failure rate (73.5%). Craniospinal radiotherapy in localized malignant NGGCT could be avoided without enhancing relapses beyond the range of radiotherapy. Chemotherapy and craniospinal radiotherapy are still the gold standards for metastatic disease.^326^

### Central nervous system lymphoma

Central nervous system lymphoma can be classified into two categories: primary and secondary. Primary central nervous system lymphoma (PCNSL) is a rare but aggressive extranodal non-Hodgkin lymphoma (NHL) that impacts the CNS, including the spinal cord, brain, leptomeninges, as well as eyes. About 90% of PCNSL cases are diffuse large B-cell lymphomas (DLBCLs), while the rest are T-cell, Burkitt’s, as well as lymphoblastic and low-grade lymphomas. Currently, PCNSL accounts for approximately 2% of all primary CNS tumors,^327^ and 4–6% of extranodal lymphomas.^328^ Secondary central nervous system lymphoma (SCNSL) refers to NHL involving the CNS, which can be manifested as lymphocytic leptomenditis and epidural spinal cord compression signs. SCNSL patients have poor outcomes, and despite dramatic advances in comprehending the mutational landscape of primary diffuse large B-cell lymphoma (DLBCL), there is still a lack of genetic comparison to SCNSL.^329^

### Pathophysiology

At present, the pathogenesis of PCNSL has not been defined.^330,331^ EB virus has been detected in immunocompromised PCNSL patients, so it is believed that EBV with carcinogenic effects may be related to the pathogenesis of PCNSL, but no EB virus genomic DNA has been detected in patients with normal immune function.^332,333^ Evidence suggests PCNSL exhibits an overlap of differentiation, expressing germinal center biomarkers such as B cell lymphoma 6 (BCL6) and activation markers such as cyclin D2 and MUM1/Interferon Regulatory Factor 4 (IRF4).^334^ There is a high frequency of single nucleotide variants and copy number alterations in PCNSL. It has been reported that myeloid differentiation primary response 88 (MYD88) and CD79B are involved in both activated B-cell-like (ABC) and germinal center B-cell-like (GCB) subtypes of PCNSL. The MYD88 and CD79B gene mutation together leads to the B cell receptor signaling pathway activation to promote the development and progression of PCNSL.^335^ MYD88 missense mutations result in constitutive activation of the TLR pathway,^336^ while CD79B alteration activates the BCR pathway.^337^ Caspase activation and recruitment domain 11(CARD11) mutations activate both pathways downstream,^337^ while tumor necrosis factor alpha-induced protein 3 (TNFAIP3) alterations can cause pathways to lose inhibition.

**Fig. 6:**
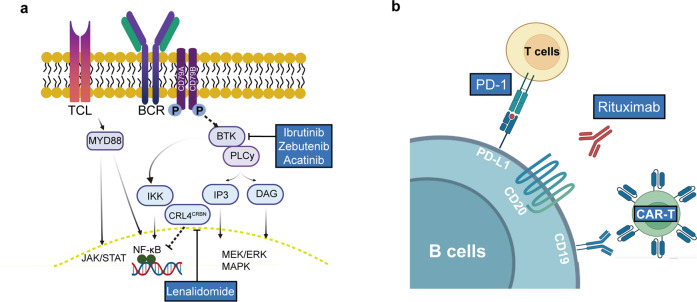
Schematic drawing of the genes, cellular interactions, or signaling pathways targeted by therapeutic strategy for PCNSL. Multi-signaling pathways associated with the malignant B cells’ development and activation are implicated in PCNSLs. Specific targets in B cells and the Toll-like receptor (TLR) pathway, the BCR signaling pathway, and immune microenvironment regulation were exploited for precision therapy. (a) BCR- and TLR-mediated signaling transduction in PCNSL. (b) Immuno-and-targeted therapies in the TME for PCNSL. Created with BioRender.com (https://biorender.com) and Reactome pathway database (https://reactome.org/)

Many studies indicated that the tumor microenvironment is also an essential factor in PCNSL development. Tumor-associated macrophages (TAMs) have been revealed to be responsible for promoting cancer invasion, proliferation, and immunosuppression in PCNSL cells. The quantification of TAMs may function in prognosis.^338^ Additionally, TAMs overexpress PD-L1, suggesting that immunotherapy may be effective against them. Activation of the Janus kinase 2 (JAK2)/STAT3 pathway leads to the gene transcription that is implicated in cellular angiogenesis, proliferation, and survival. Meanwhile, the STAT3 gene is found to be expressed in various types of cancer, including PCNSL.^339^ Amplification of chromosome 9p24.1 leads to elevated expression of PD-L1 and PD-L2, while PD-L1 and PD-L2 can participate in the immune evasion and regulatory mechanisms of PCNSL.^340^ Additionally, somatic hypermutation (SHM) may lead to PCNSL pathogenesis and may offer a rationale for immunotherapy. Major molecular alterations and related pathways in PCNSL were shown in Fig. 6.

### Therapies for PCNSL

Treatment for PCNSL has developed over the last 40 years, while the optimal treatment for PCNSL has not been determined. The advent of high-dose (HD) methotrexate (HD-MTX) therapy has ameliorated PCNSL prognosis.^341^ At present, HD-MTX-based chemotherapy is recognized as the first-line treatment. Although the initial HD-MTX-based treatment has a high response rate, more than 50% of initial responders relapse.^342^ PCNSL is sensitive to radiotherapy, and whole-brain radiotherapy (WBRT) can consolidate the response to chemotherapy. Nevertheless, WBRT-related delayed neurotoxicity results in neurocognitive impairment, particularly in elderly patients.^343^ PCNSL is a DLBCL phenotypic subtype, while the standard DLBCL regimens such as prednisone, doxorubicin, vincristine, cyclophosphamide, and variations, are ineffective in this disease, and the overall effect remains unsatisfactory.^344^ Other effective approaches consist of rituximab,^345^ TMZ,^346^ as well as autologous stem-cell transplantation (ASCT).^346^ Besides, for novel drugs against PCNSL, including those targeting the B-cell receptor signaling pathway, clinical trials are being conducted. In the mid-to-late 1990s of the 20th century, surgical resection is not regarded as the standard of treatment for PCNSL. There may be a small proportion of patients with large lesions, acute symptoms, as well as signs of brain herniation who will benefit from tumor debulking. Some grow diffusely, and surgical treatment cannot make PCNSL patients benefit, thus enhancing the risk of neurological deficits.^347^

With the rapid development of precision medicine, targeted therapy is hopeful for further improving the prognosis of PCNSL. It is estimated that 90% of PCNSL is diffuse large B cell lymphoma (DLBCL), which expresses universal B cell markers (CD19, CD20, CD79a). Rituximab is a chimeric monoclonal antibody targeting CD20 and has significant activity in CD20-positive DLBCL. At present, the efficacy of rituximab in PCNSL therapy is still controversial. In phase II randomized controlled clinical trial (IELSG 32), rituximab treatment improved survival in newly diagnosed PCNSL patients.^348^ However, a recent phase III large randomized controlled clinical study (HOVON 105/ALLG NHL 24) involving 200 PCNSL patients showed that the addition of rituximab to the treatment scheme of newly diagnosed PCNSL patients did not improve the efficacy of PCNSL patients.^349^ Although the outcomes of the two randomized controlled clinical studies (RCT) were inconsistent, the latest NCCN guidelines recommended rituximab as a first-line combination therapy for PCNSL.

In recent years, targeted therapy and immunotherapy have brought promise for PCNSL treatment. Targeted therapy of PCNSL mainly focuses on Bruton tyrosine kinase (BTK) inhibitors and anti-CD20 monoclonal antibodies. Immunotherapy mainly focuses on immunomodulators, PD-1, and CAR-T. As mentioned earlier, rituximab is a cell–surface protein expressed on mature B cells, while not in neurons or glial cells. The rituximab efficacy in systemic B-cell lymphoma has been well-defined, and regimens containing rituximab have become a better choice for this setting.

Referring to the findings of a phase I study rituximab alone or combined with MTX intraventricular administration was safe and effective in PCNSL.^350^ At present, HD-MTX combined with rituximab is still the first-line treatment for PCNSL. A clinician can inject rituximab intrathecally if PCNSL has cerebrospinal fluid dissemination and HD-MTX is intolerable but must pay attention to the patient’s health. BTK is a member of the non-receptor tyrosine kinase Tec family, which is mainly expressed in various stages of B cell growth, mediates a series of cellular pathways, including B cell antigen receptor (BCR), and has an important influence on the viability, differentiation, and apoptosis of B cells.^351^ In a phase I clinical trial, ibutinib, as the first generation of BTK inhibitor, significantly improved the survival of patients.^352^ In addition, the findings of the Phase Ib clinical trial showed that the combined regimen of Ibutinib and HD-MTX (±rituximab) was well tolerated, with 80% of the total response rate.^353^ The Phase I/II clinical trial of Tirabrutinib, acting as a second-generation BTK inhibitor, exhibited a certain effect in recurrent/refractory PCNSL.^354^ Other second-generation BTK inhibitors, such as Zebutenib and Acatinib, have got the approval of the FDA for treating recurrent/refractory mantle cell lymphoma, showing higher efficacy and safety than Ibutinib, and are expected to be developed as the preferred anti-tumor drug to replace Ibutinib. Nowadays, many prospective clinical studies on the treatment of recurrent/refractory PCNSL with second-generation BTK inhibitors are in progress.

Lenalidomide is an immunomodulator that directly or indirectly inhibits tumors through unique immunomodulation. In a phase I clinical trial, lenalidomide resented marked mono-drug activity in patients with recurrent/refractory PCNSL.^355^ The Phase II REVLRI clinical trial evaluated the clinical efficacy of lenalidomide in recurrent/refractory PCNSL. The total response rate was 39%, with a median PFS of 7.8 months and a median OS of 17.7 months.^356^ Considering the above encouraging results, NCCN guidelines recommend the use of lenalidomide alone or in conjunction with rituximab for recurrent or refractory PCNSL.

Checkpoints play a part in the human immune system, acting as brakes to prevent excessive activation of T cells from causing inflammatory reactions. PD-1 and its ligand, PD-L1/PD-L2, exert functions in checkpoint pathways. Researchers stated that PD-L1 expression is upregulated in PCNSL.^357^ In a retrospective study,^358^ Navuximab was used in patients with relapsed refractory PCNSL/PLT. Four patients obtained complete remission, one patient got partial remission, and the median PFS was 9 months (7–11 months). In light of the above encouraging results, NCCN guidelines recommend lenalidomide alone or combined rituximab as a treatment regimen for recurrent/refractory PCNSL.

Autologous stem cell transplantation is a novel treatment model, and its effectiveness in curing patients with recurrent and high-risk systemic lymphoma has reached clinical recognition and has been applied to the treatment of PCNSL. It is particularly effective for young patients with recurrence but can result in higher treatment-related mortality rates in elderly patients. CAR-T cells targeting CD19 have become the leading engineered T-cell therapy approach for relapsed/refractory B-cell non-Hodgkin lymphoma.^343^ CAR-T therapy can achieve a complete remission rate of more than 50% in relapsed and refractory DLBCL. Nevertheless, due to the neurotoxicity of CAR-T therapy, patients with CNS involvement were excluded from clinical trials.^359^ Abramson et al.^360^ reported that a case of SCNSL treated with CAR-T showed that the lesion disappeared. This research result firstly proves that CAR-T cells can penetrate the blood–brain barrier and achieve the therapeutic response of the CNS, which brings a new dawn for CAR-T to treat PCNSL. A schematic representation of the actions of therapies on these signaling cascades and immune regulation is given in Fig. 6.

## Conclusion

Since the implementation of high throughput data analysis, the comprehension of the molecular profile of brain tumors has continued to evolve rapidly. The fifth edition of the WHO classification of CNS tumors in 2021 has incorporated many advanced molecular alterations into the diagnostic standards. These multitudes of cancer-specific genetic alterations, including receptor kinases and their downstream signaling partners, cell cycle regulation, telomere maintenance, and chromatin organization, and the further effects on tumor etiology have reformed the conception of clinical management and prognosis, providing new insights into the transformation of clinical trials. However, the current failure of several targeted agents, especially for GBM, illustrates that CNS tumors do not only rely on a single pathway-driven targeted therapy. Future treatment may be improved in the following ways: 1) the combination strategies of multiple targeted drugs and immunotherapeutic approaches have been proven to be efficacy against brain tumors, especially for recurrent/progressive patients, and could be the trend of treatment management in the future; 2) the limited scale of participation and specific patient groups indicates the necessity of performing more larger and multicenter clinical trials to assess efficacy and safety; 3) developing more effective drug delivery system to overcome the blood–brain barrier, such as nano-drug or extracellular vesicle-based drug delivery system; 4) performing a genetic/precision medical treatments based on the genomics technologies.
